# Identification and Quantification of 29 Active Substances by HPLC–ESI-MS/MS in Lyophilized Swine Manure Samples

**DOI:** 10.3390/molecules28010216

**Published:** 2022-12-26

**Authors:** Carolina Nebot, Alejandra Cardelle-Cobas, Ignacio García-Presedo, Ewelina Patyra, Alberto Cepeda, Carlos M. Franco

**Affiliations:** 1 Department of Analytical Chemistry, Nutrition and Bromatology, Faculty of Veterinary Medicine, Universidade de Santiago de Compostela, 27002 Lugo, Spain; 2 Asociación de Desenvolvemento Rural Mariñas-Betanzos Reserva de Biosfera Mariñas Coruñesas e Terras do Mandeo, Abegondo, 15318 Coruña, Spain; 3 Department of Hygiene of Animal Feedingstuffs, National Veterinary Research Institute, 24-100 Pulawy, Poland

**Keywords:** swine, drugs, feces, manure, non-invasive method, HPLC–MS/MS

## Abstract

Veterinary drugs are frequently employed to treat and prevent diseases in food-producing animals to improve animal health and to avoid the introduction of microorganisms into the food chain. The analysis of the presence of pharmaceutical residues in animal manure could help to evaluate the legal and illegal practices during food production without harming the animals and to correctly manage manure when it is going to be applied as a fertilizer. This article describes a method for the simultaneous analysis of 29 active substances, mostly antibiotics and antiparasitic agents. Substances were extracted from lyophilized manure with a methanol:McIlvaine solution and analyzed with HPLC–ESI-MS/MS and a C18 HPLC column. The method was validated following European guidelines, the achieved trueness was between 63 and 128% (depending on the analytes), and the linearity was between 100 and 1500 µg/kg. The applicability of the method was demonstrated in 40 manure samples collected from pig farms where tetracycline was quantified in 7.5% of the samples. These results show the viability of this non-invasive method for the control of the legal and illegal administration of pharmaceuticals in food-producing animals.

## 1. Introduction

Food of animal origin is produced around the world, and animals involved in this type of production include cattle, sheep, goats, swine, poultry, and equines [1]. These animals, like humans, have diseases and need to be treated to avoid death which leads to economic losses for farmers, and, more importantly, to avoid the introduction of food pathogens in the food chain. Therefore, inspections and animal treatments are vital for consumers’ safety and human health. Veterinary treatments in food-producing animals are always conducted and controlled by veterinarians within the European Union, who choose the treatment [2]. Depending on the diseases and number of animals, medicines may be administrated in a variety of forms, including injections, tablets, creams, ointments, lotions, and sprays. For large groups of animals, pharmaceuticals are administrated through medicated feed or water. A wide range of drugs can be administrated to food-producing animals, including non-steroidal anti-inflammatory agents, antibiotics, and coccidiostats [1]. Drugs are metabolized and excreted through feces or urine as metabolites or in the unmetabolized form, and the percent of excretion of the unmetabolized form is variable and dependent on the drugs. For example, 66% of the initial dose of the antibiotic sulfachloropyridazine is excreted unchanged [3]. On the other hand, only 11% of the initial dose of sulfamethoxazole is excreted unchanged [4].

The analysis of the presence of active substances such as antibiotics in swine manure is relevant from two points of view. First is the food safety perspective, as it is a non-invasive way to control the legal or illegal administration of veterinary drugs to food-producing animals, as samples can be easily taken from the floor without stressing or damaging animals. Food of animal origin is controlled with different monitoring plans to ensure food safety; however, the analysis of manure is an interesting way to curtail illegal practices. On the other hand, the presence of active substances in manure needs to be controlled from an environmental point of view, as manure is employed as a natural fertilizer for farmland or grassland [5,6] and pharmaceuticals are transferred from the manure to soils and the water, thus contaminating rivers, lakes, and drinking water sources [7,8]. The concentration of antibiotics in swine manure has been shown to be between a few μg/kg and several hundred mg/kg [9,10] depending on the location of the farm, the farm size, and the treatment applied to the animals. One of the most relevant problem of the environmental presence of antibiotics is the increased development of bacteria with resistance genes. In a study conducted in the Netherlands where feces samples from pigs and cattle were analyzed, antibiotics were detected in more than 50% of the samples, and 34% of the samples contained more than one antibiotic, with those from the groups of tetracyclines and sulfonamides being most frequently detected [10].

Few articles on analytical method for the analysis of antibiotic in feces samples could be found in the literature because most research has focused on contaminated matrices such as water, soil, or food. Additionally, manure analysis could require different steps due to the complexity of the studied matrix, and reported methods include laborious extraction protocols [11] including the use of ultrasonic-assisted extraction [12,13,14,15,16], microwave-assisted extraction [17,18,19], and solid-phase extraction, which is the most popular method for matrix clean-up [20,21,22]. Regarding the detection of veterinary drugs high-performance liquid chromatography combined with tandem mass spectrometry is considered the best choice due to its high selectivity and sensitivity [11,23,24,25,26,27].

Even if a few methods have been reported in the literature for the analysis of active substances in animal feces samples, more reliable methods are required to control the presence of these substances in swine manure to avoid the introduction of antibiotics into the food chain or the environment; these methods will also help to reduce the illegal use of drugs in food-producing animals. Therefore, the objective of this work was to present an analytical tool based on HPLC–MS/MS for the identification and quantification of 31 active substances in swine manure. Table 1 compiles the compound, therapeutic class, CAS Number, molecular weight, and chemical formula of the selected pharmaceuticals.

## 2. Results and Discussion

### 2.1. Optimization of the LC–MS/MS Method

The selected compounds were detected with a mass spectrometer (MS) employing electrospray ionization (ESI) in the negative or positive mode depending on the analyte. For correct analyte identification, precursor and product ions, as well as the electrospray ionization (ESI) mode, were optimized by infusing standard solutions of each compound at 1 μg/L. Even though the samples matrix was manure, it was related to food, so Regulation 2021/808 [28] was employed as a guideline for method optimization and validation. MS optimization was achieved for most compounds; even though the employed MS has very good features for most compounds, response for coccidiostats (decoquinate, maduramicin, monesin, narasin, nicarbazin, robenidine and sarafloxacin, and salinomycin), were not the same as those previously achieved with other equipment [29,30], therefore theirs detection was discarded.

**Figure 1:**
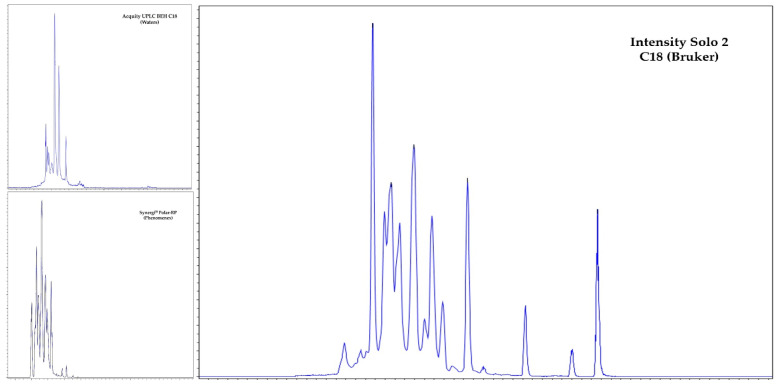
Total ion chromatograms (TICs) of the selected pharmaceuticals separated on different HPLC columns.

For the chromatographic separation of the analytes, three HPLC columns were tested; ACQUITY UPLC BEH C_18_ from Waters (Milford, USA), Intensity Solo 2 C18 from Bruker (Bremen, Germany), and Synergi Polar 5 um from Phenomenex (California, USA). Based on previously developed methods, the mobile phase was selected to be a combination of a gradient mode of water acidified with 0.1% of formic acid (mobile phase A) and acetonitrile acidified with 0.1% of formic acid (mobile phase B). The three tested columns were C18-packed, but their integration with the same analytes was different. The peak shape of mefenamic acid had a more gaussian shape with the Bruker and Phenomenex columns than with the Waters columns, and the opposite was observed for sulfamethizole. Regarding retention time (Rt), compounds eluted fastest with the Phenomenex column because it is shorter than the others. The difference in Rt varied from 0.5 min for danofloxacin to 2.9 min for mefenamic acid. Based on resolution, better peak shapes, peak high, and back pressure, the Intensity Solo HPLC column from Bruker was chosen as the most versatile column. Figure 1 shows the total ion chromatograms (TICs) of the three tested columns. As unsatisfactory chromatograms were obtained for amoxicillin, azithromycin, cefuroxime, colistin, flumethasone, griseofulvin, spectinomycin and sulfamethizole, these pharmaceuticals were not included in the final analysis. The other two columns, C18 from Waters and Phenomenex, showed similar chromatography for the discarded pharmaceuticals, but it is important to note that both of them permit the correct identification of more than 10 different compounds, as previously reported by other researchers [29,30,31,32,33]. Other columns available in the market and employed for antimicrobial detection in manure and feces samples include Nucleosil C_18_ HD [34] and Kinetex C_18_ [35]. The chromatographic performance of the HPLC method used in this study was initially investigated with a standard solution containing all selected pharmaceuticals at 100 ng/mL in mobile phase A. Replicate injections of various volumes (3, 5, 10, 15 and 20 µL) were performed to investigate repeatability and to avoid the introduction of a high volume of the sample matrix in order to obtain a good limit of detection for the selected drugs. The best results were achieved with 15 µL of injection. The reliable confirmation of the analytes was achieved with Rt and two MRM transitions from one parent and two product ions [28]. Table 2 compiles the Rt, MRM transition, and collision energy values of each analyte.

### 2.2. Extraction Procedure

**Figure 2:**
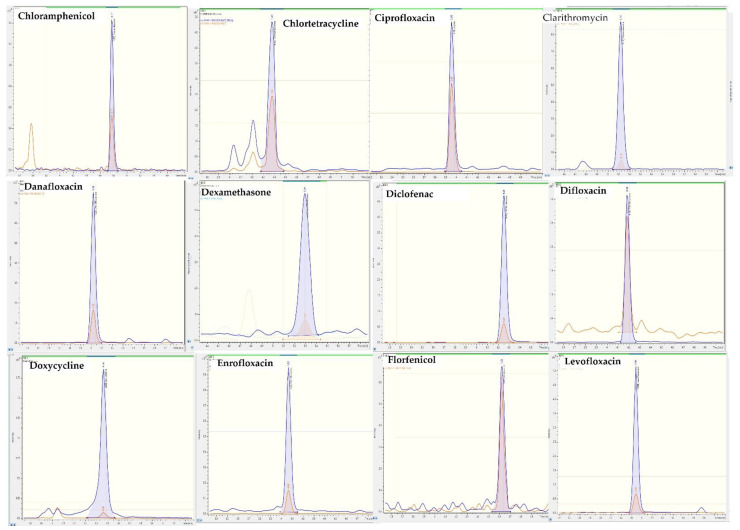

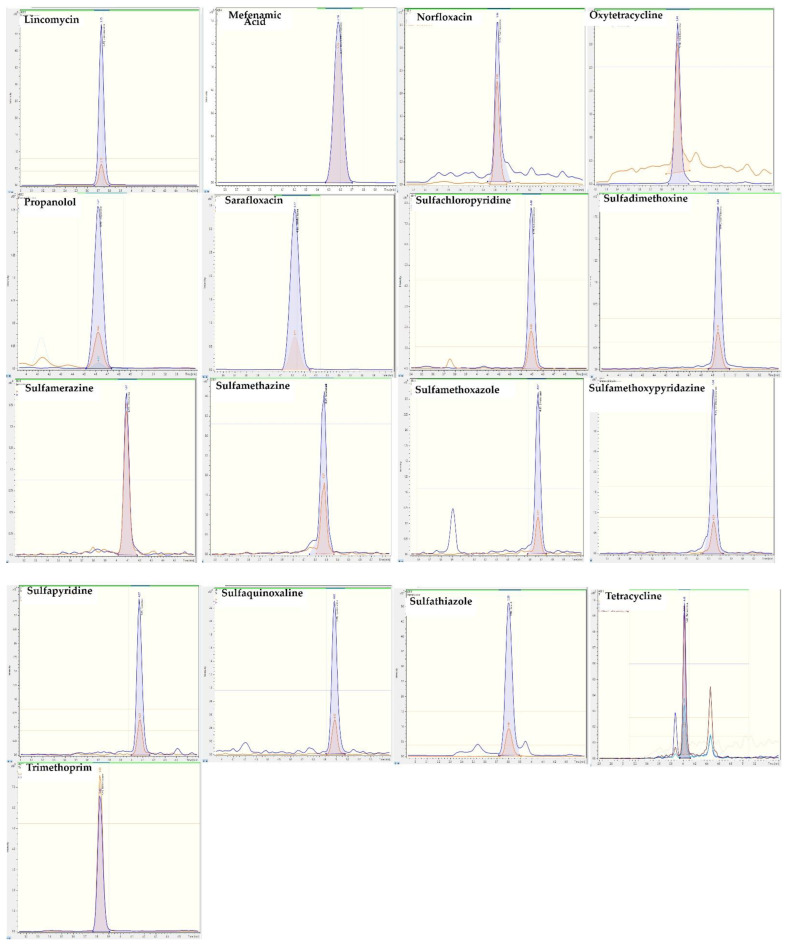
MRM chromatograms of pig feces sample fortified 29 veterinary drugs at the concentration level 400 μg/kg.

The analysis of pharmaceuticals in animal feces and manure can be difficult because it requires a complex matrix with a high level of organic matter. The primary objective of this research was to present a non-invasive analytical tool for organization related to food safety to control the administration of active substance in swine production The presented method was also aimed to be simple, inexpensive, and easy to apply in the laboratory, with reproducible results. Previously, pressurized liquid extraction enabled the extraction of toltrazuril, an antiparasitic, and its metabolites from manure collected from a piglet near Copenhagen [36]. The same technique was employed by Hansen et al. (2011) [37], who identified 10 hormones in pig manure, and by Wang et al. (2020), who extracted 33 antibiotics and 37 pesticides from livestock and poultry excrement samples [38]. Argüeso-Mata and collaborators (2021) combined two different extraction processes, dispersive solid-phase extraction and compact solid-phase extraction, to extract 21 analytes from different groups of antimicrobials such as macrolides, tetracyclines, β-lactams, sulfonamides and fluoroquinolones [39]. Approaches with QuEChERS [40] and normal solid-phase extraction with cartridges have also been reported [41]. The optimized method of extraction presented in this study does not require any material related to solid-phase extraction or pressurized liquid extraction as it employs a solvent of extraction mixture of methanol and a McIlvaine buffer. The use of this buffer combined with an organic solvent or followed by solid-phase extraction previously showed satisfactory results for the extraction of veterinary drugs from value matrices including baby food [42], feed [43] and soil [44]. One remarkable extraction protocol was described by Melekhina et al. (2021), who identified 63 veterinary drugs from various classes (sulfonamides, amphenicols, nitroimidazoles, β-lactams, macrolides, lincosamides, tetracyclines, quinolones and pleuromutilins) in chicken meat [45]. However, the protocol requires a purification step with hypercrosslinked polystyrene. This is the main advantage of the method presented here, as it only needs 10 mL of an extraction solvent. Before extraction, samples needed to be lyophilized to reduce the water content and to achieve a lower limit of detection. A total of 27 active ingredients in swine manure were satisfactorily extracted with the final extraction protocol, which was a combination of simple and short consecutive steps: a mixture of manure and the extraction solvent, sonication, agitation, centrifugation, and filtration, followed by a chromatographic method based on HPLC–MS/MS; this method enabled the correct identification and quantification of the studied compounds. Figure 2 shows MRM transition of each pharmaceutical of a matrix-matched sample spiked with pharmaceuticals at 400 µg/kg

### 2.3. Method Validation

The entire procedure of extraction and HPLC–MS/MS analysis was validated with matrix-matched calibration samples. Validation parameters evaluated included linearity, precision under repeatability and reproducibility conditions, accuracy, sensitivity, specificity, and matrix effects. The results are shown in Table 2.

On each day of validation, a calibration curve was built with eight matrix-matched lyophilized manure samples spiked with all selected analytes at concentrations from 0 to 1500 µg/kg. The coefficient of determination (R^2^) obtained for each compound on each day was 0.97 or higher, indicating good linearity. Precision under repeatability (n = 6, one day) and reproducibility conditions (n = 18, three days) showed a relative standard deviation (RSD%) of less than 20% for most compounds; out of 27, lincomycin showed the highest RSD of 34%, 30%, and 20% at 200, 400 and 600 µg/kg, respectively.

Accuracy, as defined in Regulation 808/2021, was evaluated with six replicate samples showing a close agreement between the spiked level and accepted true reference value; employing the calibration curve build on that day showed that the accuracy was between 80 and 120%. Additionally, the specificity of the method was tested by processing and analyzing 20 replicate samples with different drugs at the same concentration (400 µg/kg) and without drugs.

The potential effect of the matrix on the drug concentration calculation was also evaluated by comparing the response of the instrument to the compounds dissolved in a solvent to the response to a matrix-matched sample. In these manure samples, the matrix was complex and had a high level of interference from inorganics such as Ca, Mg, and other minerals that could form chelates; tetracyclines and other organic compounds compete with the selected pharmaceuticals in terms of extraction efficiency. These interferences not only could reduce the recoveries but also they could amplify or lower the signal response.

Matrix effects were calculated, as indicated in Regulation 2021/808, by dividing the signal of a matrix-matched sample by the signal of a standard solution at the same concentration. A result below 100% indicated ion suppression, and a result above 100% indicated ion enhancement.

The matrix effect is the effect that a matrix can have on a drug concentration calculation. It was evaluated in this study by comparing the response of the instrument to the compounds dissolved in a solvent to the response to a matrix-matched sample. In this case, feces were found to affect pharmaceutical concentration by interfering with the extraction and reducing its efficiency. The feces matrix could also interfere with the signal response by amplifying or lowering it and consequently increasing or reducing the calculated concentration. The matrix factor (MF) for each drug was calculated as the peak area of a matrix-matched standard against the peak area of a standard solution. The results are summarized in Table 2. In general, MF values were around one except for mefenamic acid, diclofenac, and lincomycin, which had values of 1.6, 1.7, and 1.4, respectively. The RSD of the MF, calculated as the mean of the MF obtained for the concentration range from LOD to 2000 ng/g, was below 20% in all cases, which is a satisfactory value according to Regulation 2021/808.

### 2.4. Application to Feces Samples

Pharmaceuticals were only detected in 4 manure samples out of 40, representing 7.5% of the analyzed samples. The compounds that were detected were doxycycline and oxytetracycline. The detection of doxycycline and oxytetracycline was an unexpected result since the animals were not treated with these substances and their concentration in the lyophilized samples did not exceed 7 mg/kg, which could indicate animal treatment at the previous stage of production. Since the treatments at the piglet weaning phase were unknown, the proposed explanation is plausible.

It is also important to highlight that even though some animals were treated with Florken and Pulmoval, no florfenicol residues were detected in the feces. Even though two samples were collected for each batch of pigs, florfenicol treatment was conducted just after the collection of the first sample and one month before the collection of the second sample. Therefore, residues of florfenicol in the animals were slowly eliminated after the treatment. For the specific case of pigs and florfenicol, the withdrawal period is 15 days.

Likewise, it should be noted that fecal analysis is a non-invasive method that allows for the detection of the illegal and legal administration of drugs to food -producing animals. The analysis of this type of sample is not a common practice for food-producing animals even though it can be used to obtain satisfactory results with a low limit of detection when lyophilization is applied to samples. Most publications on the drug analysis of animal feces, such as the work carried out by Sengeløv et al. (2003), Holzel et al. (2013), Joy et al. (2013), and Pu et al. (2018) [46,47,48,49], have focused on the environmental point of view and the impact of applying manure as a fertilizer, especially on the development of bacteria with resistance genes. Considering the results obtained within this research project and all the benefits observed for animals and farmers, the analysis of drugs in fecal samples for the detection of legal or illegal practices during animal production should be more common and standardized since it allows for control without harming animals.

## 3. Materials and Methods

### 3.1. Chemicals and Reagents

Amoxicillin, azithromycin, cefuroxime, chloramphenicol, chlortetracycline, ciprofloxacin, clarithromycin, colistin, danafloxacin, decoquinate, dexamethasone, diclofenac, difloxacin, doxycycline, enrofloxacin, erythromycin, florfenicol, flumethasone, griseofulvin, ibuprofen, levofloxacin, lincomycin, maduramicin, mefenamic acid, monesin, narasin, nicarbazin, norfloxacin, oxytetracycline, paracetamol, propranolol, robenidine, sarafloxacin, salinomycin, spectinomycin, sulfachloropyridine, sulfadiazine, sulfadimethoxine, sulfamerazine, sulfamethasone, sulfamethoxazole, sulfamethoxypyridazine, sulfapyridine, sulfaquinoxaline, sulfathiazole, tetracycline, trimethoprim, and tylosin with a purity above 98% were bought from Sigma-Aldrich (St. Louis, MO, USA). Anhydrous citric acid, trichloroacetic acid (TCA), ethylenediaminetetraacetic acid disodium salt (EDTA), and disodium hydrogen phosphate dehydrate were also purchased from Sigma-Aldrich (St. Louis, MO, USA). Acetonitrile (ACN), methanol (MeOH) (HPLC grade ≥ 99%), and formic acid (purity > 99% for analysis) were obtained from Acros Organics (Geel, Belgium). Purified water, with a resistivity higher than 18.0 MU, was prepared in the laboratory with a Milli-Q system from Millipore (Burlington, MA, USA).

### 3.2. Preparation of Reagents and Standard Solutions

Water, ACN, or MeOH were employed as solvents to prepare the standard solutions of the selected pharmaceuticals. First, an accurately weighed (±0.1 mg) amount of pharmaceutical, 10 or 20 mg measured with an analytical balance (Ohaus, Greifensee, Switzerland), was transferred into a 25 mL amber volumetric flask. The final concentration of each stock solution depended on each pharmaceutical’s solubility. The different stock solutions were mixed to obtain a 5 µg/mL working standard solution of each pharmaceutical. All solutions were stored at −20 °C for a minimum period of one month.

Mobile phases A and B were prepared by adding 500 μL of formic acid to ~400 mL of Milli-Q water (mobile phase A) or acetonitrile (mobile phase B), respectively. The volume was finally set to 500 mL with the corresponding solvent to achieve a final formic acid concentration of 0.1% in each case.

A McIlvaine buffer solution was prepared by mixing citric acid (615.4 mL at 0.1 M) with disodium hydrogen phosphate (385 mL at 0.2 M). NaOH or HCl was used to adjust the pH. Once the pH was 4, EDTA (37.2 g) was added to a 1 L McIlvaine buffer solution and stored at 8 °C for one month. The final extraction solution was a mixture of methanol and McIlvaine–EDTA (70:30), which was prepared for each day of extraction.

### 3.3. Equipment

Swine manure samples were analyzed with the following equipment: an RSLAB-9 rotatory shaker (Rogo Sampaic, Wissous, France); a Minishaker model MS2 vortex mixer (IKA, Staufen, Germany); an Eppendorf model 5910 R centrifuge (Eppendorf, Hamburg, Germany); an Intensity Solo 2 C18 90 Å HPLC column, 8 µm, 2.1 × 100 mm (Bruker, Bremen, Germany); an Acquity UPLC BEH C18 130 Å HPLC column, 1.7 µm (Waters, Milford, MA, USA); and a Synergi™ Polar-RP 100 Å HPLC column, 5 µm, 2.1 × 50 mm (Phenomenex, Torrance, CA, USA). After extraction, pharmaceuticals were analyzed on with Elute UHPLC system and a triple quadrupole EVOQ LC-TQ mass spectrometer, both from Bruker (Bremen, Germany). The whole system was controlled with tqControl version 2.0.0 from Bruker (Bremen, Germany), and HPLC without MS was controlled with EDM version 1.2 (1.2.34.0) from Bruker (Bremen, Germany).

### 3.4. Swine Manure Samples Extraction

Samples were lyophilized and stored in a freezer before drug extraction. Two grams of lyophilized swine manure was accurately weighed into a 50 mL falcon tube. Each batch of samples (n = 20) was simultaneously extracted with 8 matrix-matched control samples; these lyophilized samples were spiked with pharmaceuticals in doses of 0, 100, 200, 400, 600, 800, 1000 and 1500 µg/kg. Then, 10 mL of an extraction solvent (MeOH:McIlvaine–EDTA; 70:30, *v*/*v*) was added to each tube, and samples were vortexed for 10 s, shaken in a rotatory shaker for 30 min at room temperature, and centrifuged at 4500 rpm for 15 min at 8 °C. The final extracts were filtered through a syringe filter (Acrodisc Waters, MA, USA) and transferred to an HPLC amber vial.

Before enacting the final extraction protocol, which yielded the best recoveries and signal responses for most compounds, various conditions related to the extraction method were investigated. The tested conditions included the extraction efficiency of ACN, MeOH, and water at different percentages and in different combinations. QuEChERS extraction with the use of a mixture of water and an organic solvent (ACN or MeOH) combined with NaCl and MgSO_4_ was also tested. Other investigated parameters were: (I) sample weight, (II) extraction solvent volume, (III) rotation time, (IV) centrifugation time and temperature, and (V) the evaporation of different sample extracts for concentration. The different conditions were tested on three replicated lyophilized samples spiked with pharmaceuticals at a dose of 600 µg/kg and on a blank sample (analyte-free). Results were evaluated with a standard calibration curve of a mixture of pharmaceuticals at 0, 10, 25, 50, 100 and 250 ng/mL.

### 3.5. LC–MS/MS Conditions

The mobile phase were mixed in a gradient mode of mobile phases A and B. The flow rate was set to 0.300 mL/min with the following gradient program: 0.0–1.0 min for 100% solvent A, 1.0–6.0 min for 10% solvent A, 6.0–6.5 min for 0% solvent A, 6.5–7.5 min for 0% solvent A, 7.5–9.0 min for 100% solvent A, and 9.0–15.0 min for 100% solvent A. The temperature of the column was maintained at 42 °C during the whole run, the sample injection volume was 15 µL, and the samples were maintained at 8 °C during the sequence analysis. For the detection of most compounds with MS analysis, the positive electrospray (ESI+) mode was employed (Table 3), except for the cases of chloramphenicol and florfenicol, where the negative ESI mode was used. Drugs were determined with two multiple reaction monitoring (MRM) runs and their Rt values. In the positive and negative modes, the electrospray voltage was 4800 V and 4500 V, respectively. During analysis, the cone temperature (300 °C), cone flow (20 psi), probe temperature (500 °C), nebulizer flow (30 psi), and exhaust gas flow (50 psi) were maintained at constant values.

### 3.6. Validation

Validation was conducted following different guidelines, particularly Regulation 2021/808 and Regulation 2002/657. Evaluated aspects of the method included signal/noise ratio (S/N), the RSD of the Rt, linearity, matrix effects, recovery, precision under repeatability (RSD_r_), and reproducibility (RSD_R_). To validate this method, analyte-free lyophilized swine manure samples were spiked with the selected drugs at doses of 0, 100, 200, 400, 600, 800, 1000 and 1500 µg/kg. For each concentration, six replicates were employed, and the experiment was repeated on three different days. The validation parameters of accuracy, matrix effect, precision, sensitivity, and linear dynamic range were determined for the 31 target analytes.

### 3.7. Swine Manure Collection

Swine manure samples were collected by the veterinarian involved in the project. Once collected, the samples were kept in a sterilized container, stored in a portable fridge, and sent to the laboratory for analysis. Once in the laboratory, samples were subject to lyophilization and stored at −20 °C until analysis, which was conducted within three months after collection. The sample collection and method were conducted as part of a project entitled “Reducción de la adición de antibióticos en la dieta de animales de porcino en ciclo industrial”, in which the main objective was to design a production system based on feeding and management in order to promote good animal health in the last 3 months of animals’ lives (fattening phase) by applying different strategies related to the systems of animal production, with the final objective of not administrating antimicrobials in the final stage of animal production.

## 4. Conclusions

The present article describes the validation and application of an HPLC–MS/MS method for the identification and quantification of 29 drugs in swine manure. The method was satisfactorily employed for the control of the administration of antimicrobials to pigs in the three last months of food production. A total of 40 samples were analyzed, and only four samples showed the presence of antimicrobials in the group of tetracyclines. The results indicated that the presented method could be satisfactorily applied during swine production without harming or stressing the animals, and antimicrobials detected in samples when the animals are treated with antibiotics. Additionally, the method is quick and inexpensive, as a low amount of organic solvents is used and the amount of generated residues is low compared with other reported methods employing SPE.

## Figures and Tables

**Table 1:** Analyte name, therapeutic class, CAS Register Number (CAS), molecular weight (MW) and chemical formula of the selected pharmaceuticals.

Compound	Therapeutic Groups	CAS	MW	Stock Solution Concentration (µg/mL)	Solvent
Amoxicillin	Antibiotic	26787-78-0	365.4	800	Methanol
Azithromycin	Antibiotic	83905-01-5	749.03	800	Methanol
Cefuroxime	Antibiotic	55268-75-2	424.38	800	Methanol
Chloramphenicol	Antibiotic	56-75-7	323.13	1000	Methanol
Chlortetracycline	Antibiotic	57-62-5	478.88	1000	Methanol
Ciprofloxacin	Antibiotic	85721-33-1	331.34	400	Water:Methanol (3:1)
Clarithromycin	Antibiotic	81103-11-9	747.96	400	Methanol
Colistin	Antibiotic	1066-17-7	1155.4	800	Methanol
Danofloxacin	Antibiotic	112398-08-0	357.38	800	Methanol
Decoquinate	Antiparasitic Agent	18507-89-6	417.5	100	Methanol
Dexamethasone	Corticosteroids	50-02-2	392.5	800	Methanol
Diclofenac	Anti-Inflammatory	15307-86-5	296.15	800	Methanol
Difloxacin	Antibiotic	98106-17-3	399.4	400	Methanol
Doxycycline	Antibiotic	564-25-0	444.44	1000	Methanol
Enrofloxacin	Antibiotic	93106-60-6	359.4	800	Methanol
Erythromycin	Antibiotic	114-07-8	733.9	800	Methanol
Florfenicol	Antibiotic	73231-34-2	358.2	1000	Methanol
Flumethasone	Glucocorticoid	2135-17-3	410.5	800	Methanol
Griseofulvin	Fungistatic Agent	126-07-8	352.8	400	Methanol
Ibuprofen	Nonsteroidal Anti-inflammatory	15687-27-1	206.28	800	Methanol
Levofloxacin	Antibiotic	100986-85-4	361.37	800	Methanol
Lincomycin	Antibiotic	154-21-2	406.54	800	Methanol
Maduramicin	Antiparasitic Agent	84878-61-5	934.2	800	Methanol
Mefenamic Acid	Anti-Inflammatory	61-68-7	241.28	400	Methanol
Monesin	Antiparasitic Agent	17090-79-8	670.9	800	Methanol
Narasin	Antiparasitic Agent	555134-13-9	765.0	400	Methanol
Nicarbazin	Antiparasitic Agent	330-95-0	426.4	800	Dimethyl Sulfoxide
Norfloxacin	Antibiotic	70458-96-7	319.33	800	Methanol
Oxytetracycline	Antibiotic	79-57-2	460.44	1000	Methanol
Paracetamol	Nonsteroidal Anti-Inflammatory	103-90-2	151.16	800	Methanol
Propranolol	Beta Blocker	525-66-6	259.34	800	Methanol
Robenidine	Antiparasitic Agent	25875-51-8	334.2		Methanol
Sarafloxacin	Antibiotic	98105-99-8	385.36	400	Methanol
Salinomycin	Antiparasitic Agent	53003-10-4	751.0		Methanol
Spectinomycin	Antibiotic	1695-77-8	332.35	400	Water:H^+^
Sulfachloropyridazine	Antibiotic	80-32-0	284.73	50	Methanol
Sulfadiazine	Antibiotic	68-35-9	250.28	50	Methanol
Sulfadimethoxine	Antibiotic	122-11-2	310.33	50	Methanol
Sulfamerazine	Antibiotic	127-79-7	264.31	50	Methanol
Sulfamethazine	Antibiotic	57-68-1	278.33	50	Methanol
Sulfamethoxazole	Antibiotic	723-46-6	253.28	50	Methanol
Sulfamethoxypyridazine	Antibiotic	80-35-3	280.3	50	Methanol
Sulfapyridine	Antibiotic	144.83-2	249.29	50	Methanol
Sulfaquinoxaline	Antibiotic	59-40-5	300.34	50	Methanol
Sulfathiazole	Antibiotic	72-14-0	255.32	50	Methanol
Tetracycline	Antibiotic	60-54-8	444.43	1000	Methanol
Trimethoprim	Antibiotic	738-70-5	290.32	800	Methanol
Tylosin	Antibiotic	1401-69-0	916.1	800	Methanol
Amoxicillin	Antibiotic	26787-78-0	365.4	800	Methanol
Azithromycin	Antibiotic	83905-01-5	749.03	800	Methanol
Cefuroxime	Antibiotic	55268-75-2	424.38	800	Methanol
Chloramphenicol	Antibiotic	56-75-7	323.13	1000	Methanol
Chlortetracycline	Antibiotic	57-62-5	478.88	1000	Methanol
Ciprofloxacin	Antibiotic	85721-33-1	331.34	400	Water:Methanol (3:1)
Clarithromycin	Antibiotic	81103-11-9	747.96	400	Methanol
Colistin	Antibiotic	1066-17-7	1155.4	800	Methanol
Danofloxacin	Antibiotic	112398-08-0	357.38	800	Methanol
Decoquinate	Antiparasitic Agent	18507-89-6	417.5	100	Methanol
Dexamethasone	Corticosteroids	50-02-2	392.5	800	Methanol
Diclofenac	Anti-Inflammatory	15307-86-5	296.15	800	Methanol
Difloxacin	Antibiotic	98106-17-3	399.4	400	Methanol
Doxycycline	Antibiotic	564-25-0	444.44	1000	Methanol
Enrofloxacin	Antibiotic	93106-60-6	359.4	800	Methanol
Erythromycin	Antibiotic	114-07-8	733.9	800	Methanol
Florfenicol	Antibiotic	73231-34-2	358.2	1000	Methanol
Flumethasone	Glucocorticoid	2135-17-3	410.5	800	Methanol
Griseofulvin	Fungistatic Agent	126-07-8	352.8	400	Methanol
Ibuprofen	Nonsteroidal Anti-Inflammatory	15687-27-1	206.28	800	Methanol
Levofloxacin	Antibiotic	100986-85-4	361.37	800	Methanol
Lincomycin	Antibiotic	154-21-2	406.54	800	Methanol
Maduramicin	Antiparasitic Agent	84878-61-5	934.2	800	Methanol
Mefenamic Acid	Anti-Inflammatory	61-68-7	241.28	400	Methanol
Monesin	Antiparasitic Agent	17090-79-8	670.9	800	Methanol
Narasin	Antiparasitic Agent	555134-13-9	765.0	400	Methanol
Nicarbazin	Antiparasitic Agent	330-95-0	426.4	800	Dimethyl Sulfoxide
Norfloxacin	Antibiotic	70458-96-7	319.33	800	Methanol
Oxytetracycline	Antibiotic	79-57-2	460.44	1000	Methanol
Paracetamol	Nonsteroidal Anti-Inflammatory	103-90-2	151.16	800	Methanol
Propranolol	Beta Blocker	525-66-6	259.34	800	Methanol
Robenidine	Antiparasitic Agent	25875-51-8	334.2		Methanol
Sarafloxacin	Antibiotic	98105-99-8	385.36	400	Methanol
Salinomycin	Antiparasitic Agent	53003-10-4	751.0		Methanol
Spectinomycin	Antibiotic	1695-77-8	332.35	400	Water:H+
Sulfachloropyridazine	Antibiotic	80-32-0	284.73	50	Methanol
Sulfadiazine	Antibiotic	68-35-9	250.28	50	Methanol
Sulfadimethoxine	Antibiotic	122-11-2	310.33	50	Methanol
Sulfamerazine	Antibiotic	127-79-7	264.31	50	Methanol
Sulfamethazine	Antibiotic	57-68-1	278.33	50	Methanol
Sulfamethoxazole	Antibiotic	723-46-6	253.28	50	Methanol
Sulfamethoxypyridazine	Antibiotic	80-35-3	280.3	50	Methanol
Sulfapyridine	Antibiotic	144.83-2	249.29	50	Methanol
Sulfaquinoxaline	Antibiotic	59-40-5	300.34	50	Methanol
Sulfathiazole	Antibiotic	72-14-0	255.32	50	Methanol
Tetracycline	Antibiotic	60-54-8	444.43	1000	Methanol
Trimethoprim	Antibiotic	738-70-5	290.32	800	Methanol
Tylosin	Antibiotic	1401-69-0	916.1	800	Methanol

**Table 2:** Matrix effects, RSD matrix effects (RSD_ME_), precision under repeatability (RSDr) and reproducibility (RSDR) conditions, trueness, and correlation coefficient (R^2^) achieved at different concentrations for each pharmaceutical.

Compound	Concentration	Matrix Effects	RSD_ME_ (%)	RSD_r_	RSD_R_	Trueness	a	b	R^2^
	(µg/kg)			(%) (n = 6)	(%) (n = 18)	(%) (n = 18)			
Chloramphenicol	200	0.9	7.5	13	11	118	3300.7	70.4	0.971
	400			29	5	110			
	600			9	7	117			
Chlortetracycline	200	1.3	15.8	20	13	141	22,883.9	3167.6	0.981
	400			27	13	110			
	600			11	12	117			
Ciprofloxacin	200	1.0	10.5	12	14	98	53,985.2	5468.9	0.986
	400			21	14	113			
	600			3	14	107			
Clarithromycin	200	0.5	13.0	21	5	107	68,053.0	906.3	0.966
	400			41	7	111			
	600			8	16	136			
Danafloxacin	200	0.6	8.1	7	18	99	16,787.8	4841.5	0.978
	400			19	12	104			
	600			5	11	106			
Dexamethasone	200	0.0	11.3	21	11	100	82.1	100.9	0.972
	400			13	6	119			
	600			16	11	97			
Diclofenac	200	0.4	2.9	10	12	110	49,795.8	3404.6	0.998
	400			26	11	102			
	600			10	9	102			
Difloxacin	200	0.3	3.0	9	18	113	30,438.3	2226.7	0.977
	400			20	11	109			
	600			5	15	102			
Doxycycline	200	2.8	8.4	11	16	95	1,294,454.7	14660.6	0.998
	400			18	6	103			
	600			6	17	108			
Enrofloxacin	200	1.2	9.0	18	10	90	236,205.7	7664.0	0.982
	400			12	8	117			
	600			14	9	92			
Florfenicol	200	0.9	9.0	18	10	111	2720.1	37.8	0.975
	400			24	9	118			
	600			13	6	139			
Levofloxacin	200	0.5	14.4	13	16	102	93,029.5	3682.9	0.971
	400			23	5	102			
	600			3	14	98			
Lincomycin	200	5.5	2.1	34	16	70	178,170.3	43379.3	0.977
	400			28	15	66			
	600			20	10	74			
Mefenamic Acid	200	2.3	19.1	24	16	118	273,977.7	13018.5	0.994
	400			38	12	82			
	600			8	14	95			
Norfloxacin	200	0.5	1.1	12	8	101	210,771.6	2414.2	0.969
	400			22	9	110			
	600			4	9	114			
Oxytetracycline	200	0.5	1.1	8	20	124	183,941.1	2705.8	0.977
	400			24	13	84			
	600			13	10	109			
Propranolol	200	0.4	3.8	13	20	118	77,055.7	1916.3	0.991
	400			21	17	118			
	600			10	7	126			
Sarafloxacin	200	0.7	3.6	7	18	109	96,223.4	4575.7	0.98
	400			19	10	111			
	600			6	15	96			
Sulfachloropyridine	200	1.0	3.7	10	22	113	239,127.4	5107.6	0.984
	400			25	13	123			
	600			6	11	144			
Sulfadimethoxine	200	0.8	3.6	6	15	117	480,417.2	13543.4	0.975
	400			23	11	113			
	600			5	11	115			
Sulfamerazine	200	1.8	3.6	5	22	117	67,678.2	4582.8	0.979
	400			24	12	108			
	600			3	11	116			
Sulfamethazine	200	1.5	2.5	6	25	132	645,449.5	13561.1	0.974
	400			23	13	122			
	600			4	12	125			
Sulfamethoxazole	200	0.9	5.5	12	22	111	185,586.8	5934.0	0.974
	400			28	18	106			
	600			8	9	128			
Sulfamethoxypyridazine	200	1.6	3.6	6	23	117	153,709.0	11111.6	0.976
	400			22	12	110			
	600			3	12	115			
Sulfapyridine	200	1.2	4.9	5	22	111	90,339.4	8788.6	0.983
	400			27	13	112			
	600			5	11	123			
Sulfaquinoxaline	200	0.8	4.6	6	21	115	216,109.5	4361.8	0.985
	400			34	14	118			
	600			5	13	137			
Sulfathiazole	200	0.5	5.4	6	21	116	70,093.4	6651.6	0.97
	400			25	18	117			
	600			18	4	125			
Tetracycline	200	0.1	5.2	7	18	107	16,481.5	1369.9	0.997
	400			28	6	109			
	600			5	17	115			
Trimethoprim	200	0.3	5.2	8	19	111	58,128.0	6639.5	0.975
	400			27	12	119			
	600			5	11	121			

**Table 3:** Retention time (Rt) and multiple reaction monitoring (MRM) runs 1 and 2 employed for pharmaceutical identification.

Compound	Rt (min)	RSD of Rt (%)	MRM 1	MRM 2
Chloramphenicol	4.82	0.5	(−) 323.0 > 152.0 [14.0 V]	(−) 323.0 > 194.1 [9.0 V]
Chlortetracycline	4.43	0.2	(+) 479.0 > 462.0 [15.0 V]	(+) 479.0 > 444.0 [22.0 V]
Ciprofloxacin	4.00	0.4	(+) 332.2 > 314.1 [16.0 V]	(+) 332.2 > 231.0 [32.0 V]
Clarithromycin	5.17	0.3	(+) 749.0 > 158.0 [25.0 V]	(+) 749.0 > 116.0 [50.0 V]
Danafloxacin	4.07	0.3	(+) 358.0 > 340.0 [25.0 V]	(+) 358.0 > 255.0 [35.0 V]
Dexamethasone	5.34	0.2	(+) 393.0 > 373.0 [7.0 V]	(+) 393.0 > 354.6 [10.0 V]
Diclofenac	6.29	0.2	(+) 296.0 > 215.0 [15.0 V]	(+) 296.0 > 151.0 [60.0 V]
Difloxacin	4.23	0.2	(+) 386.0 > 299.0 [25.0 V]	(+) 386.0 > 299.0 [25.0 V]
Doxycycline	4.52	1.7	(+) 445.0 > 428.0 [15.0 V]	(+) 445.0 > 154.0 [30.0 V]
Enrofloxacin	4.11	3.9	(+) 360.0 > 342.1 [17.0 V]	(+) 360.0 > 286.0 [31.0 V]
Florfenicol	4.66	1.1	(−) 358.0 > 185.0 [15.0 V]	(−) 358.0 > 338.0 [5.0 V]
Levofloxacin	3.98	1.1	(+) 362.0 > 261.0 [30.0 V]	(+) 362.0 > 179.0 [40.0 V]
Lincomycin	3.73	0.6	(+) 407.3 > 126.2 [22.0 V]	(+) 407.3 > 359.2 [12.0 V]
Mefenamic Acid	6.61	0.2	(+) 242.0 > 223.8 [15.0 V]	(+) 242.0 > 209.0 [27.0 V]
Norfloxacin	3.96	0.3	(+) 320.0 > 302.0 [15.0 V]	(+) 320.0 > 276.0 [15.0 V]
Oxytetracycline	3.96	0.4	(+) 461.0 > 426.0 [20.0 V]	(+) 461.0 > 443.0 [10.0 V]
Paracetamol	3.58	2.9	(+) 152.3 > 110.0 [23.0 V]	(+) 152.3 > 92.7 [23.0 V]
Propranolol	4.67	1.4	(+) 260.0 > 116.0 [20.0 V]	(+) 260.0 > 154.5 [20.0 V]
Sarafloxacin	4.27	0.2	(+) 400.0 > 299.0 [30.0 V]	(+) 400.0 > 382.0 [30.0 V]
Sulfachloropyridine	4.52	0.2	(+) 285.0 > 156.0 [11.0 V]	(+) 285.0 > 108.0 [18.0 V]
Sulfadiazine	3.74	1.6	(+) 251.1 > 156.0 [12.0 V]	(+) 251.1 > 108.0 [19.0 V]
Sulfadimethoxine	4.97	0.2	(+) 311.0 > 156.0 [20.0 V]	(+) 311.0 > 108.0 [18.0 V]
Sulfamerazine	4.05	1.3	(+) 265.0 > 156.0 [16.0 V]	(+) 265.0 > 172.0 [16.0 V]
Sulfamethazine	4.25	0.6	(+) 279.0 > 186.0 [15.0 V]	(+) 279.0 > 156.0 [15.0 V]
Sulfamethoxazole	4.64	0.2	(+) 254.0 > 156.0 [11.0 V]	(+) 254.0 > 92.0 [18.0 V]
Sulfamethoxypyridazine	4.27	0.2	(+) 281.0 > 156.0 [13.0 V]	(+) 281.0 > 92.0 [24.0 V]
Sulfapyridine	3.93	4.1	(+) 250.0 > 156.0 [13.0 V]	(+) 250.0 > 92.0 [23.0 V]
Sulfaquinoxaline	4.98	0.2	(+) 301.0 > 156.0 [15.0 V]	(+) 301.0 > 92.0 [25.0 V]
Sulfathiazole	3.86	3.1	(+) 256.0 > 156.0 [12.0 V]	(+) 256.0 > 92.0 [22.0 V]
Tetracycline	4.08	5.9	(+) 445.4 > 410.0 [20.0 V]	(+) 445.4 > 427.0 [15.0 V]
Trimethoprim	3.88	0.4	(+) 291.0 > 123.0 [20.0 V]	(+) 291.0 > 230.0 [24.0 V]

## Data Availability

Not applicable.

## Sample Availability

Samples of the compounds are not available from the authors.
